# Commentary: Mentorship by new graduate registered nurses – lessons for supportive learning environments

**DOI:** 10.1177/17449871261475974

**Published:** 2026-08-31

**Authors:** Marie Jönsson

**Affiliations:** RN, PhD, Kristianstad University, Kristianstad, Sweden

**Commentary to:** Nursing students’ experiences of mentorship by NGRNs on placement: a qualitative descriptive study (Sood et al., 2026a)

## Summary of the article

Given the global nursing shortage and the increasing reliance on new graduate registered nurses (NGRNs) as mentors, there is a critical need to understand how their mentorship influences nursing students’ clinical learning experiences. This study, ‘Nursing students experiences of mentorship by new graduate registered nurses (NGRNs) on placement: A qualitative descriptive study’ by Sood et al. (2026a), aimed to explore pre-registration nursing students’ experiences of mentorship by NGRNs. The study was conducted in an Australian University where pre-registration nursing students enrolled in a pre-registration Bachelor of Nursing (BN) programme. Participants were mentored by an NGRN with a mean age of 29.5 years. The participants were recruited using purposive and snowball sampling. Data were collected with individual semi-structured Zoom interviews with 12 (2 males) pre-registration nursing students and the interviews lasted between 15 and 30 minutes. The data were analysed guided by Braun and Clarke’s thematic analysis. The interview study showed three themes and five subthemes. The first theme highlights that mentorship by NGRNs fostered a strong sense of belonging by creating psychologically safe, welcoming and empathetic learning environments where students felt valued, supported and confident to participate in clinical learning. The second theme suggests that NGRNs contribute not only through emotional support but also through effective pedagogical practices. By promoting collaborative learning, allowing time for reflection and gradually fostering independence, they create learning experiences that strengthen students’ confidence, clinical reasoning and professional development. The third theme highlights that the effectiveness of mentorship by NGRNs is constrained by organisational and contextual factors, including workload, staffing shortages, limited clinical experience and insufficient support for mentors.

## Connection to nursing theory, practice and research

The findings resonate with my research on person-centred leadership, which emphasises the importance of creating trusting and supportive work environments where people feel respected, valued and empowered to learn. Although the study focuses on mentorship by newly graduated registered nurses, my clinical experience and experience of supervising nursing students support the view that mentorship and leadership are closely interconnected components of the registered nurse’s professional role, a perspective that is also reflected in the broader nursing literature (Abdul-Rahim et al., 2025).

Both require the ability to build trusting relationships, facilitate learning and development and create care environments that enable others to grow. Consequently, the mentoring behaviours identified in this study closely reflect key characteristics of person-centred leadership.

The first theme, describing how approachable, empathetic and non-judgemental mentors fostered nursing students’ sense of belonging, aligns with work by McCance and McCormack (2025). It demonstrates the importance of being in relation, which is fundamental to person-centred cultures, and highlights that developing interpersonal skills, knowing self and having clarity of values and beliefs are nursing prerequisites. Similar findings on the importance of relationships have been reported in my own research, where both first-line managers and registered nurses described building trust by being available; and respectful relationships as prerequisites for learning, engagement and quality care, and also the importance of being a role model for others (Jönsson et al., 2025a, 2025b).

An important finding is the emphasis on reflective questioning and structured reflection as strategies to promote nursing students’ learning, confidence, and clinical reasoning (Sood et al., 2026a). This resonates with findings from my own research on person-centred leadership in residential care facilities, where both first-line managers and registered nurses described reflection as a central leadership practice to enable person-centred care (Jönsson et al., 2025a, 2025b). Rather than providing answers, leaders facilitated reflective dialogue that encouraged health care staff to critically examine their practice, develop professional judgement and engage in shared learning.

The third theme highlights that even highly motivated mentors cannot provide optimal learning experiences without organisational support. Workload pressures, staffing shortages and insufficient senior support limited the ability of new graduated registered nurses to mentor effectively (Sood et al., 2026a). These findings are consistent with our studies of person-centred leadership in residential aged care, which showed that person-centred leadership depends on organisational conditions that enable practice (Jönsson et al., 2025a). This reflects McCance and McCormack’s (2025) Person-centred Nursing Framework, where supportive organisational systems are prerequisites for person-centred practice. Consequently, effective mentorship relies not only on the leadership and supervisory capabilities of individual registered nurses but also on leadership at organisational and managerial levels that creates the conditions necessary for registered nurses to mentor, lead and supports nursing students effectively.

Similar conclusions were reached in a recent integrative review also by Sood et al. (2026b) whose findings suggest that effective mentorship requires mentors to provide consistent support, foster a sense of belonging within the clinical environment and deliver constructive feedback to students. However, several factors within the clinical setting hinder effective mentorship, including inadequate staffing levels, heavy workloads, time constraints, limited interest in mentoring and nurses’ competing professional responsibilities.

Developing new graduate nurses as mentors therefore requires not only pedagogical preparation but also leadership strategies that create psychologically safe workplaces, provide protected time for learning and support reflective practice.

## Practical implications: who can benefit from this research?

The findings suggest that healthcare organisations can strengthen clinical learning by preparing NGRNs for mentoring roles, providing structured supervision, allocating protected time for teaching and fostering psychologically safe learning environments.

For managers, the study provides evidence that assigning students to NGRNs should be accompanied by organisational support rather than being viewed as an informal responsibility. Decisions regarding workload allocation, staffing and mentor preparation may directly influence the quality of students’ clinical learning experiences (Sood et al., 2026a).

Although conducted in acute care settings (Sood et al., 2026a), the findings are likely to be relevant across healthcare contexts where newly qualified nurses supervise students, including community care, primary care and residential aged care. These findings may be particularly relevant in residential aged care, where workforce shortages often result in newly graduated registered nurses taking on supervisory responsibilities early in their careers. Creating supportive organisational cultures may therefore benefit both student learning and workforce retention.
